# Anxiety and orienting of gaze to angry and fearful faces

**DOI:** 10.1016/j.biopsycho.2007.07.005

**Published:** 2007-10

**Authors:** Karin Mogg, Matthew Garner, Brendan P. Bradley

**Affiliations:** School of Psychology, University of Southampton, Highfield, Southampton SO17 1BJ, UK

**Keywords:** Anxiety, Angry faces, Fearful faces, Attentional bias, Eye movements

## Abstract

Neuroscience research indicates that individual differences in anxiety may be attributable to a neural system for threat-processing, involving the amygdala, which modulates attentional vigilance, and which is more sensitive to fearful than angry faces. Complementary cognitive studies indicate that high-anxious individuals show enhanced visuospatial orienting towards angry faces, but it is unclear whether fearful faces elicit a similar attentional bias. This study compared biases in initial orienting of gaze to fearful and angry faces, which varied in emotional intensity, in high- and low-anxious individuals. Gaze was monitored whilst participants viewed a series of face-pairs. Results showed that fearful and angry faces elicited similar attentional biases. High-anxious individuals were more likely to direct gaze at intense negative facial expressions, than low-anxious individuals, whereas the groups did not differ in orienting to mild negative expressions. Implications of the findings for research into the neural and cognitive bases of emotion processing are discussed.

## 1 Introduction

Research into the cognitive and neural mechanisms underlying emotion processing indicates neural circuitry, involving subcortical and cortical structures (including the amygdala and prefrontal cortex), which is responsible for detecting threat-related cues in the environment and triggering a variety of cognitive, behavioural and physiological responses, in particular, attentional vigilance (LeDoux, 1996; Davis and Whalen, 2001). Neuroimaging studies show that the amygdala, which plays a central role in this circuitry, is reactive to threat-related cues, such as fearful faces (e.g., Whalen et al., 1998). Individual differences in the operation of this threat-processing system may underlie individual differences in vulnerability to anxiety and anxiety disorders (Davis and Whalen, 2001).

From a clinical perspective, cognitive models of anxiety also assume the existence of a threat-processing system and propose that anxiety is characterized by cognitive biases (in particular, in stimulus evaluation and selective attention) which favour the processing of threat cues. These biases are proposed to be responsible for individual differences in anxiety vulnerability (e.g., Beck and Emery, 1985; Williams et al., 1997; Mogg and Bradley, 1998). Cognitive models, such as Beck and Emery's (1985), are important because they provide the basis for cognitive-behaviour therapy (CBT), which aims to remove these threat-processing biases and is an effective treatment for many anxiety disorders (e.g., Ballenger, 1999). Research into cognitive models of anxiety indicates that anxious individuals have an enhanced attentional bias for threat cues, compared with non-anxious individuals, and that this bias operates in early aspects of processing (e.g., review by Mogg and Bradley, 1998).

In order to understand the functional properties of this postulated threat-processing system, it is necessary to clarify the type of stimuli to which it is sensitive. Neuroimaging research typically indicates that the amygdala is more reactive to fearful than angry faces (e.g., Whalen et al., 2001; Blair et al., 1999; Davis and Whalen, 2001; but see Yang et al., 2002). Davis and Whalen (2001) suggested that fearful faces elicit more amygdala activity because they are ambiguous (i.e. they signify the presence of danger, but do not provide information about its source) and that the threat-processing system is more reactive to ambiguous (or indirect) threat cues because it is designed to promote attention to stimuli which require more detailed processing in order to determine appropriate responding (e.g., escape or approach). Neuroimaging evidence has been reported supporting the hypothesis of enhanced processing of ambiguous threat cues, as indexed by amygdala responses to angry and fearful faces (Adams et al., 2003). Thus, according to Davis and Whalen's (2001) theoretical view, fearful faces should be particularly effective in capturing attention and in eliciting vigilance.

Biases in visuospatial orienting to threat faces have been investigated using visual-search, visual-probe and eye-movement paradigms. Visual-search studies require participants to search arrays of faces for discrepant emotional expressions and results have indicated faster detection of schematic angry faces, relative to positive faces (e.g., Öhman et al., 2001; Fox et al., 2000). In the visual-probe task, pairs of faces (e.g., angry face and neutral face of the same individual) are briefly presented, followed by a probe stimulus (e.g., small dot) to which the participant responds. Response times (RTs) to probes reflect the allocation of attention to the faces, as RTs are typically faster to probes which appear in attended, rather than unattended, locations. Visual-probe studies provide evidence of enhanced attentional biases for angry faces in individuals with high non-clinical anxiety, and in anxious patients (e.g., Bradley et al., 1998, 1999; Mogg et al., 2004). They also indicate that the attentional bias increases as the intensity of the angry expression increases (Wilson and MacLeod, 2003). Eye-movement methodology has extended this research, as it provides a direct and ecologically-valid measure of visual orienting, and further indicates that anxious individuals have a greater bias to direct their gaze initially towards angry faces (relative to neutral or happy faces), compared with non-anxious individuals (Bradley et al., 2000; Mogg et al., 2000), which suggests that the attentional bias for threat operates at a relatively early stage of visual processing and guides eye-movements. However, there has been little research using these paradigms to assess biases in visual orienting towards fearful faces. For example, one study using a visual-search task suggested that angry, but not fearful, faces preferentially attract attention (Williams et al., 2005), whereas another study using a visual-probe task indicated an attentional bias for fearful faces that had been perceptually degraded (i.e. low spatial frequency; Holmes et al., 2005). Neither study examined the influence of anxiety.

Consequently, the main aim of the present study was to compare biases in initial orienting of gaze towards fearful versus angry faces in high- and low-anxious individuals. A second aim was to examine the effect of manipulating the emotional intensity of the faces (cf. Blair et al., 1999; Wilson and MacLeod, 2003) on attentional responses. Neuroimaging research (Davis and Whalen, 2001) predicts that fearful faces should elicit stronger vigilance responses than angry faces. In addition, cognitive models of anxiety predict that high-anxious individuals should show greater biases in initial orienting of gaze towards both angry and fearful faces, than low-anxious individuals. Furthermore, if biases in initial orienting to threat-related cues are primarily a function of the affective salience of the stimuli (Mogg and Bradley, 1998), then these biases in orienting should increase as the intensity of the fearful and angry expressions increases.

## 2 Method

### 2.1 Participants

Participants were undergraduates who were screened on the trait version of the State-Trait Anxiety Inventory (STAI; Spielberger, 1983); those with trait anxiety scores of 40 or less were allocated to the low anxiety group, and those scoring 50 or more to the high anxiety group. Additional selection criteria were fluency in spoken English and visual acuity within normal limits. Nine volunteers did not complete the study due to equipment calibration problems and six participants had excessive missing eye-movement data (described later). The final sample comprised 28 participants (3 M, 25 F) in the low-anxious group, and 21 (3 M, 18 F) in the high-anxious group.

### 2.2 Materials and apparatus

**Fig. 1:**
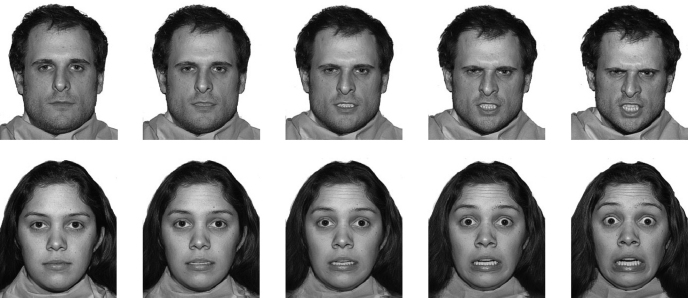
Example of continuum for angry (top row) and fearful (bottom row) facial expressions.

Twenty-four face stimuli were selected from the NimStim Set of Facial Expressions (http://www.macbrain.org/faces/).^1^ They consisted of angry, fearful and neutral prototypical expressions posed by eight models (four female and four male numbered: 01f, 03f, 07f, 08f, 21m, 23m, 27m, 34m). For each model and type of facial expression (fearful or angry), the emotional face was blended with the neutral face to produce a continuum comprising five exemplars of increasing emotional intensity: i.e. neutral prototype (0%), three morphed expressions (25%, 50% and 75%) and the emotional prototype (100%); see Fig. 1. The preparation of the morphed faces used Gryphon Morph v2.5 software (Gryphon Software Corporation, 1994). Each emotional face (i.e. 25%, 50%, 75% or 100% exemplar of a fearful or angry face) was paired with the neutral expression (0%) of the same model to create 64 face pairs for use in the attentional task.

The task was administered using MEL2 software (Schneider, 1995), Pentium 450 MHz PC, 15 in. VGA monitor and MEL2 response box. Eye-movements were monitored with 120 Hz infrared pan/tilt eye tracking system (Model 504, Applied Scientific Laboratories, Bedford, Massachusetts) and E5000 software (Applied Science Group, 2000) which was run on a Pentium 333 MHz PC. Testing was conducted in a dimly lit room.

### 2.3 Procedure

Participants completed a visual acuity check and were seated 1 m from the monitor, with the eye tracking camera positioned 50 cm in front of them, below the right eye. The equipment was calibrated by displaying the numbers 1 to 9 on the screen in a 3 × 3 array and recording the direction of gaze whilst participants looked at each number in turn.

Each trial of the attentional task started with a central fixation-cross shown for 1000 ms, followed by a pair of pictures presented side by side for 500 ms. The pictures measured 90 mm × 110 mm, with their inner edges 45 mm apart. Immediately after the offset of the picture-pair, a probe (pair of dots : or ..) was presented in the position of one of the preceding pictures (visual angle of 7.7° between two probe positions) until a manual response. Participants were asked to press one of two keys as quickly as possible to indicate the type of probe. Inter-trial interval varied randomly between 750 and 1250 ms. Participants were instructed to keep their head still throughout the task and to look at the fixation-cross at the start of each trial. Eye-movement data were recorded from the onset of the fixation-cross until the manual response. There were eight practice trials followed by two blocks, each consisting of two buffer trials and 256 experimental trials, with a short rest-break between the blocks. Across the whole task, the 64 face pairs were presented eight times, balanced for emotional face location and probe location (left vs. right). Trials were presented in a new random order for each participant. If eye calibration quality deteriorated during the task, the task was briefly interrupted to repeat the calibration procedure.

After the attentional task, participants completed three face rating tasks: forced-choice discrimination, anger and fear ratings, which were included to check the effectiveness of the manipulation of emotional intensity. Each task presented the faces from the attentional task, one at a time, in random order. Each trial consisted of a central fixation-cross (500 ms), followed by a face and rating scale, displayed until a keyboard response. In the forced-choice discrimination task, participants indicated whether each face was angry or fearful. In the anger and fear rating tasks, they rated each face for how angry or fearful it appeared on a scale ranging from 0 (not at all) to 8 (extremely). Finally, they completed questionnaires including the state and trait versions of the STAI, and short-form Social Desirability Scale (SDS; Strahan and Gerbasi, 1972; the latter was included because defensiveness can have a confounding effect on measures of anxiety and attentional bias, e.g., Eysenck, 1997).

### 2.4 Data preparation

#### 2.4.1 Eye-movement data

These were prepared using the Eyenal Data Analysis Program (Applied Science Group, 2000). Direction of gaze was measured in degrees approximately once every 8 ms. If eye-movements were stable within 1° of visual angle for 100 ms or more, this was classified as a fixation to that position. Initial fixations to the pictures were calculated if the following criteria were met: (i) gaze was fixated in the central region before picture onset, (ii) fixations occurred at least 100 ms after picture onset, and (iii) fixations were directed at either the left or right picture (>1.3° wide of central fixation position on the horizontal plane). Six participants had excessive missing data (85% or more of trials lacked identifiable fixations), so were excluded from all analyses. The amount of missing data did not vary as a function of anxiety group, *F*(1, 47) < 1.7, emotional face type, emotional face intensity, or their interaction, *F*(3, 141) <1.5, all *p*'s > .2. Gaze-direction bias scores were obtained for each participant and each level of intensity of fearful and angry faces by calculating the number of trials in which the first shift of gaze was towards the emotional face, as a proportion of the number of trials in which the initial shift in gaze was made to either the emotional or neutral face (bias scores > .5 reflect an orienting bias towards the emotional face; .5 = no bias).

#### 2.4.2 RT data

Data from error trials (2%) and RTs <200 ms or >1250 ms (1%) were excluded. The groups did not differ in missing data or overall mean RT, *t*s < 1. An inverse transformation was applied to the RT data to reduce the influence of skewness and outliers (Ratcliff, 1993). Attentional bias scores were calculated using a standard formula (e.g., Bradley et al., 1998), for each participant and each level of intensity of fearful and angry faces, by subtracting the mean RT when the emotional face and probe were in the same location from the mean RT when the emotional face and probe were in different locations. RT bias scores were corrected (reverse sign and multiply by 10^6^) so that they would be easier to comprehend, i.e. positive values of bias scores reflect an attentional bias for emotional faces, relative to neutral faces (0 = no bias).

Kolmogorov–Smirnov tests showed that the distributions of RT bias scores and gaze-direction scores were within normal limits.

## 3 Results

### 3.1 Group characteristics

The high- and low-anxious groups differed significantly in trait and state anxiety (see Table 1), but not in social desirability scores, age or gender ratio.

### 3.2 Eye-movement data

**Fig. 2:**
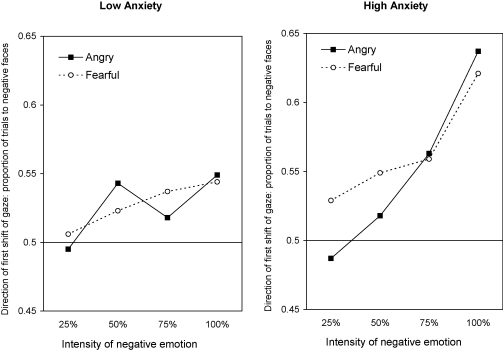
Mean proportion of trials on which the initial shift of gaze was directed towards negative rather than neutral faces, shown as a function of the type (fearful or angry) and emotional intensity of the facial expression, in the low anxiety (left panel) and high anxiety (right panel) groups.

Gaze-direction bias scores were entered into a 2 × 2 × 4 mixed design ANOVA with anxiety group (high, low) as the between-subjects independent variable (IV), and emotional face type (angry, fearful) and intensity of emotional face (25%, 50%, 75%, 100%) as within-subject IVs (see Fig. 2 for means). There were significant main effects of anxiety group, *F*(1, 47) = 4.68, *p* < .01, $\eta_{\text{p}}^{2}=.09$, and emotion intensity, *F*(3, 141) = 10.49, *p* < .01, $\eta_{\text{p}}^{2}=.18$, which were qualified by a significant anxiety group × emotion intensity interaction, *F*(3, 141) = 3.02, *p* < .05, $\eta_{\text{p}}^{2}=.06$. There were no other significant results, e.g., anxiety group × emotion intensity × type of emotional face: *F* < 1. Separate ANOVAs of bias scores from each group showed a significant main effect of emotional intensity on gaze direction in the high-anxious group, *F*(3, 60) = 9.54, *p* < .001, $\eta_{\text{p}}^{2}=.32$, but not in the low-anxious group, *F*(3, 81) = 2.04, *p* = .12, $\eta_{\text{p}}^{2}=.07$. To clarify further the anxiety group × emotion intensity interaction, the groups were compared on their bias scores at each level of intensity (averaged across angry and fearful faces).

There was no significant difference between the high- and low-anxious groups in bias scores for 25% negative faces, (*M*s = .508 vs. .500, *t* < 1, NS, *d* = .11), 50% negative faces (.533 vs. .533, *t* < 1, NS, *d* = .003), or 75% negative faces (.561 vs. .528, *t*(47) = 1.34, NS, *d* = .39). A one-sample *t*-test was used to contrast the RT bias scores against a value of .5 (which indicates no bias); results showed that, irrespective of group, participants showed no attentional bias for 25% negative faces (.504, *t* < 1, NS, *d* = .05), but a significant bias for 50% negative faces (.533, *t*(48) = 2.99, *p* < .01, *d* = .43) and also for 75% negative faces (.542, *t*(48) = 3.39, *p* < .01, *d* = .48).

For 100% negative faces, the high-anxious group showed a significantly greater bias in orienting towards them, compared with the low-anxious group (.629 vs. .547, *t*(47) = 3.21, *p* < .01, *d* = .91). One-sample *t*-tests showed a significant bias in gaze towards 100% negative faces, relative to neutral faces, in both the high-anxious (.629, *t*(20) = 5.66, *p* < .001, *d* = 1.24) and low-anxious (.547, *t*(27) = 3.26, *p* < .01, *d* = .62) groups. Thus, the bias for participants to direct their gaze initially at prototypical (100%) negative faces, was significantly stronger in the high-anxious group.

### 3.3 Manual RT data

RT bias scores were entered into a 2 × 2 × 4 mixed design ANOVA with anxiety group, type of emotional face and intensity of emotional face as IVs. There was a significant group × emotion intensity interaction, *F*(3, 141) = 2.83, *p* < .05, $\eta_{\text{p}}^{2}=.06$, and no other significant results (e.g., group × intensity × face type: *F* < 1). The pattern of significant findings corresponded to that in the eye movement data. The high- and low-anxious groups did not significantly differ in RT bias scores for 25% negative faces (−7.4 vs. 16.4, *t*(47) = 1.40, NS, *d* = .40), 50% negative faces (13.1 vs. 19.0, *t* < 1, NS, *d* = .11), or 75% negative faces (4.5 vs. 24.9, *t*(47) = 1.17, NS, *d* = .33). Contrasts of RT bias scores against zero showed that, irrespective of group, participants showed no attentional bias for 25% negative faces (6.2 vs. zero, *t* < 1, *d* = .10), a significant bias for 50% negative faces (16.4, *t*(48) = 2.12, *p* < .05, *d* = .30), and a near-significant bias for 75% negative faces (16.2, *t*(48) = 1.87, *p* < .07, *d* = .27).

For 100% negative faces, there was a significant group difference in attentional bias (*t*(47) = 2.04, *p* < .05, *d* = .60). Contrasts of the bias scores against zero showed a significant attentional bias for 100% negative faces in the high-anxious group (37.4, *t*(48) = 3.48, *p* < .01, *d* = .76), but not in the low-anxious group (4.2, *t* < 1, *d* = .07).

The RT bias and gaze-direction bias scores for 100% negative faces significantly correlated with each other (*r* = .35, *p* < .05). There were no significant associations between these two measures of attentional bias for negative faces at lower levels of emotion intensity (*r* = .11, .10 and .13 for 25%, 50% and 75% negative faces, respectively, all NS).^2^

### 3.4 Rating tasks

#### 3.4.1 Forced choice discrimination task

A 2 (anxiety group) × 2 (emotional face type) × 4 (intensity of emotional face^3^) ANOVA of the proportion of faces correctly classified as angry or fearful showed a significant main effect of intensity, *F*(3, 141) = 173, *p* < .001, $\eta_{\text{p}}^{2}=.79$, as, unsurprisingly, more intense emotional expressions were classified more accurately. Mean proportion of correctly classified negative faces with 25%, 50%, 75% and 100% intensity was .70, .94, .97 and .96, respectively. There were no other significant results (e.g., group × intensity, *F*(4, 188) = 1.26, *p* > .25; group × intensity × face type: *F* < 1).

#### 3.4.2 Anger ratings

A 2 (anxiety group) × 5 (emotion intensity) ANOVA of ratings of faces from the *anger continuum* showed only a significant main effect of intensity, *F*(4, 188) = 594, *p* < .001, $\eta_{\text{p}}^{2}=.93$, with no effects involving group, *F*s < 1. Mean anger ratings of 0%, 25%, 50%, 75% and 100% angry faces were 1.6, 2.8, 4.8, 6.2 and 7.0, respectively. Bonferroni-corrected contrasts showed that each mean differed significantly from each other, *p* < .005.

A 2 × 5 ANOVA of anger ratings of faces from the *fear continuum* showed only a significant effect of intensity, *F*(4, 188) = 11.63, *p* < .001, $\eta_{\text{p}}^{2}=.20$, with no effects involving group, *F*s < 1. Mean anger ratings of 0%, 25%, 50%, 75% and 100% fearful faces were 1.6, 1.1, 0.8, 0.8 and 0.7, respectively. Bonferroni-corrected contrasts showed that faces with fearful content (i.e. 25–100%) were rated as less angry than neutral faces, *p* < .005.

#### 3.4.3 Fear ratings

A 2 × 5 ANOVA of fear ratings of faces from the *fear continuum* showed a significant main effect of intensity, *F*(4, 188) = 515, *p* < .001, $\eta_{\text{p}}^{2}=.92$, with no effects involving group (group: *F* < 1; group × intensity: *F*(4, 188) = 1.20, NS). Mean fear ratings of 0%, 25%, 50%, 75% and 100% fearful faces were 1.3, 2.3, 4.4, 5.9, and 6.6, respectively. Bonferroni-corrected contrasts showed that each mean differed significantly from each other, *p* < .005.

A 2 × 5 ANOVA of fear ratings of faces from the *anger continuum* showed a significant main effect of emotion intensity, *F*(4, 188) = 2.72, *p* < .05, $\eta_{\text{p}}^{2}=.06$, and no effects involving group, *F*s < 1. Mean fear ratings of the 0%, 25%, 50%, 75% and 100% angry faces were 1.3, 1.5, 1.2, 1.0 and 0.9, respectively. Bonferroni-corrected contrasts did not reveal significant differences between these ratings.

## 4 Discussion

The present findings indicate that fearful and angry faces elicited similar biases in visuospatial orienting. There was a greater tendency for participants to direct gaze initially towards faces with moderate or intense threat-related facial expressions (50–100% intensity), relative to neutral faces, whereas mild threat-related facial expressions (25% intensity) did not elicit a bias in initial orienting. Moreover, visuospatial orienting to both fearful and angry faces was significantly influenced by individual differences in anxiety: high-anxious individuals showed a greater tendency to direct gaze at prototypical (100%) threat-related faces, irrespective of whether the faces depicted fear or anger. The secondary measure of attentional bias, which was obtained from manual RTs, showed a similar pattern of results to that obtained in the eye-movement data.

The enhanced attentional bias in high-anxious individuals, which was found for prototypical angry faces, is compatible with previous findings, e.g., from visual-probe or eye-movement studies (e.g., Mogg and Bradley, 1998; Mogg et al., 2000). The present results further indicate that high- and low-anxious individuals did not differ significantly in visual orienting to threat-related faces which had weaker emotional expressions. This may explain why some studies might fail to find evidence of an effect of individual differences in anxiety on attentional bias, if they use less salient exemplars of emotional facial expressions than the prototypical faces used here.

The finding that fearful and angry faces elicited a similar pattern of attentional bias is consistent with cognitive and neural models which posit the existence of a threat-processing system which modulates vigilance for potential sources of danger. Previous research using prototypical expressions suggests that this bias in visual orienting of gaze is specific to threat-related faces, as, for example, anxious patients have a greater bias to shift their gaze initially towards angry faces, but not happy or sad faces, compared with non-anxious controls (Mogg et al., 2000). Given this previous finding, the present study did not include these additional face types; moreover, these would have made the task too long and fatiguing for participants.

The finding of an equivalent pattern of attentional bias for fearful and angry faces is not consistent with expectation from some recent neuroimaging research findings (discussed earlier) which indicated that the amygdala, which is proposed to modulate attention to threat-related cues, is more sensitive to fearful than angry faces (Davis and Whalen, 2001). However, this might be resolved by considering the different component processes of attention, namely, *shifting* versus *maintenance* (LaBerge, 1995; Serences et al., 2005). Thus, the amygdala may indeed modulate attention to threat, but its level of activation may be a function of both initial orienting and maintained attention. Fearful and angry faces may have a similar capacity to attract attention initially (as indicated by the present findings), but may differ in the extent to which they *hold* attention. After initial orienting to angry faces, attention may be subsequently directed away from them, e.g., due to emotion-regulation processes (Mogg and Bradley, 1998; Rohner, 2002; Garner et al., 2006); whereas attention may be maintained longer on fearful faces if they require more detailed processing to determine appropriate responding, as suggested by Davis and Whalen (2001). This hypothesis could be addressed by both cognitive and neuroimaging studies: for example, eye-movement studies, which employ longer display times to assess the time-course of attentional responses (e.g., >1 s), could compare biases in both initial orienting and gaze dwell time for fearful versus angry faces.^4^ Neuroimaging studies could examine whether amygdala response to angry faces is positively associated with initial orienting to threat, but inversely associated with subsequent attentional avoidance; as suggested by research indicating that amygdala activity is suppressed by inhibitory influences of the prefrontal cortex, which mediates emotion-regulatory processes including attention control (Nomura et al., 2004; Ochsner and Gross, 2005). Thus, although many neuroimaging studies have focused largely on the role of the amygdala in emotion processing, it is important to consider it within the context of a broader network involving other structures (e.g., prefrontal cortex, thalamus, hippocampus, periaqueductal gray, locus coeruleus), with which it has reciprocal connections and which subserve a variety of functions, including attention, startle, escape and avoidance (Davis and Whalen, 2001; McNaughton and Corr, 2004).

The results from the face rating tasks confirmed the content validity of the morphed face stimuli, which were well-differentiated in emotion intensity. The groups did not differ in their explicit ratings of the faces, which is compatible with previous research that has also failed to reveal anxiety-related differences in ratings of emotional faces, and which might be due to demand or social desirability effects obscuring anxiety-related effects on self-report data (e.g., Wilson and MacLeod, 2003). These null results contrast with those from the attentional task which indicated that individual differences in anxiety played an important role in determining the processing of emotional stimuli, namely, in visual orienting. Thus, to clarify the neurocognitive mechanisms underlying emotion processing, it would seem helpful for neuroimaging studies to make greater use of tasks that are sensitive to individual differences in anxiety, such as attentional paradigms (e.g., Monk et al., 2006), and also to routinely assess individual differences in anxiety, given that this is an important determinant of attentional responses to threat.

## Figures and Tables

**Table 1:** Group characteristics

	Low anxiety		High anxiety		*t*(47)	*p*
	*M*	S.D.	*M*	S.D.		
STAI trait anxiety						
Screening	33.4	4.5	55.2	4.6	16.79	<.01
Test session	32.4	5.5	51.1	6.5	10.86	<.01

STAI state anxiety	35.1	9.1	46.2	8.6	4.34	<.01
Social desirability scale	4.1	2.5	3.6	1.9	0.74	NS
Age	19.4	1.9	20.1	1.9	1.27	NS

## Footnotes

1 Development of the MacBrain Face Stimulus Set was supported by the MacArthur Foundation Research Network; please contact Nim Tottenham (tott0006@tc.umn.edu) for further information.

2 Analyses of untransformed RT data did not show significant results. However, it is reassuring to note that the pattern of results from the RT data following inverse-transformation (recommended by Ratcliff, 1993) was similar to that found in the gaze-direction data. The gaze-direction data (which were of primary interest in the present study) did not require transformation prior to analyses.

3 Neutral faces (i.e. 0% emotion intensity) were excluded from this analysis because they cannot be classified as correct or incorrect judgments.

4 Another variable which may influence biases in shifting versus maintenance of attention to threat faces is the direction of gaze of the stimulus face. While this was not the focus of the present study, research suggests that attention reflexively follows the gaze-direction of others and this effect is modulated by emotional influences, including facial expression and observer's anxiety level (Mathews et al., 2003; Holmes et al., 2006). See also Adams et al. (2003) for gaze-direction effects on neural responses to threat.
